# Application across species of a one health approach to liquid sample handling for respiratory based -omics analysis

**DOI:** 10.1038/s41598-021-93839-9

**Published:** 2021-07-12

**Authors:** Anna E. Karagianni, Samantha L. Eaton, Dominic Kurian, Eugenio Cillán-Garcia, Jonathan Twynam-Perkins, Anna Raper, Thomas M. Wishart, R. Scott Pirie

**Affiliations:** 1 The Roslin Institute and Royal (Dick) School of Veterinary Studies, University of Edinburgh, Easter Bush, Midlothian, EH25 9PS UK; 2 Child Life and Health, University of Edinburgh, 20 Sylvan Place, Edinburgh, EH9 1UW UK

**Keywords:** Computational biology and bioinformatics, Immunology, Biomarkers, Medical research, Molecular medicine, Respiratory tract diseases

## Abstract

Airway inflammation is highly prevalent in horses, with the majority of non-infectious cases being defined as equine asthma. Currently, cytological analysis of airway derived samples is the principal method of assessing lower airway inflammation. Samples can be obtained by tracheal wash (TW) or by lavage of the lower respiratory tract (bronchoalveolar lavage (BAL) fluid; BALF). Although BALF cytology carries significant diagnostic advantages over TW cytology for the diagnosis of equine asthma, sample acquisition is invasive, making it prohibitive for routine and sequential screening of airway health. However, recent technological advances in sample collection and processing have made it possible to determine whether a wider range of analyses might be applied to TW samples. Considering that TW samples are relatively simple to collect, minimally invasive and readily available in the horse, it was considered appropriate to investigate whether, equine tracheal secretions represent a rich source of cells and both transcriptomic and proteomic data. Similar approaches have already been applied to a comparable sample set in humans; namely, induced sputum. Sputum represents a readily available source of airway biofluids enriched in proteins, changes in the expression of which may reveal novel mechanisms in the pathogenesis of respiratory diseases, such as asthma and chronic obstructive pulmonary disease. The aim of this study was to establish a robust protocol to isolate macrophages, protein and RNA for molecular characterization of TW samples and demonstrate the applicability of sample handling to rodent and human pediatric bronchoalveolar lavage fluid isolates. TW samples provided a good quality and yield of both RNA and protein for downstream transcriptomic/proteomic analyses. The sample handling methodologies were successfully applicable to BALF for rodent and human research. TW samples represent a rich source of airway cells, and molecular analysis to facilitate and study airway inflammation, based on both transcriptomic and proteomic analysis. This study provides a necessary methodological platform for future transcriptomic and/or proteomic studies on equine lower respiratory tract secretions and BALF samples from humans and mice.

## Introduction

The European Thoroughbred horseracing sector has an annual economic impact of €12 billion, including approximately 155,000 employees in a variety of roles (British Horseracing Authority). Racehorses commonly develop airway inflammation and/or exercise induced pulmonary haemorrhage during training, with prevalence rates as high as 70–80% and 100% respectively, resulting in a significant impact on animal welfare and the racehorse industry^1–3^. Cytological analysis of tracheal wash (TW) and bronchoalveolar lavage fluid (BALF) is the most commonly applied method of assessing lower respiratory tract health of the horse and helps to guide therapy^4,5^. The diagnostic application of this technique includes the identification of common equine airway disorders of the horse, including equine asthma and exercise induced pulmonary haemorrhage^6,7^. Tracheal wash, BALF, peritoneal fluid and blood samples are commonly obtained by equine veterinarians and analysed for diagnostic purposes^8–11^. Despite that, a limited number of equine airway derived gene expression studies have been reported in the literature, largely aimed at defining the pathogenesis of equine asthma^12–14^. In each of these studies, the airway cells were derived from BALF samples, which are more difficult to obtain due to the invasive collection procedure. In comparison, the analyses of TW samples rarely extend beyond cytology and subjective scoring of mucus content. However, recent technological advances in sample collection and processing procedures have made it possible to determine whether a wider range of analyses might be applied to TW samples. This would be advantageous as equine tracheal secretions represent a rich source of: (i) cells, (ii) host-derived microbiome, (iii) transcriptome and (iv) local environment-derived proteins; and (v) are relatively simple to collect with minimal discomfort to the horse.

Similar approaches have already been applied to a comparable sample set in humans^15,16^; namely, induced sputum. Induced sputum in humans provides a non-invasive method to sample airway biofluids that are enriched in proteins and may reveal novel mechanisms in the pathogenesis of respiratory diseases, such as asthma and chronic obstructive pulmonary disease^16,17^. For equine derived samples, the proteomic approach is novel and follows a recent upsurge of interest in proteomic analysis of human airway secretions as a highly informative non-invasive method of assessing the mechanisms associated with lung disease^15^. Application of both a transcriptomic and proteomic approach to equine TW samples has the potential to reveal novel biomarkers of early disease development or disease associated tissue remodelling. Furthermore, such investigations could provide additional criteria on which to base disease subcategorization (e.g., equine asthma) and/or identify novel therapeutic targets.

In keeping with the One-Health-concept, the horse is widely acknowledged as an attractive model for human asthma^3,18,19^, addressing needs that cannot be addressed through small animal models: mice are widely recognised as poor models of the human immune response, particularly in relation to the lung^20,21^. In addition to the important similarities between horses and humans with regard to both pathophysiology and macrophage/monocyte biology, the horse also represents an ideal source of large volumes of various biological sample types^18,19,22–25^. For example, the volume and the associated cell retrieval rates derived from horses represent at least 2–3 orders of magnitude greater than those obtained from rodent models. This is of particular relevance in light of the extremely limited availability of human derived samples.

Thus, in this study we investigated the most appropriate means of (a) isolating airway macrophages from TW and (b) maximising the yield and quality of RNA and protein from equine TW samples. Furthermore, we compared the relevance of our results to murine and paediatric samples, determining whether a similar approach might be adopted.

## Results

**Figure 1:**
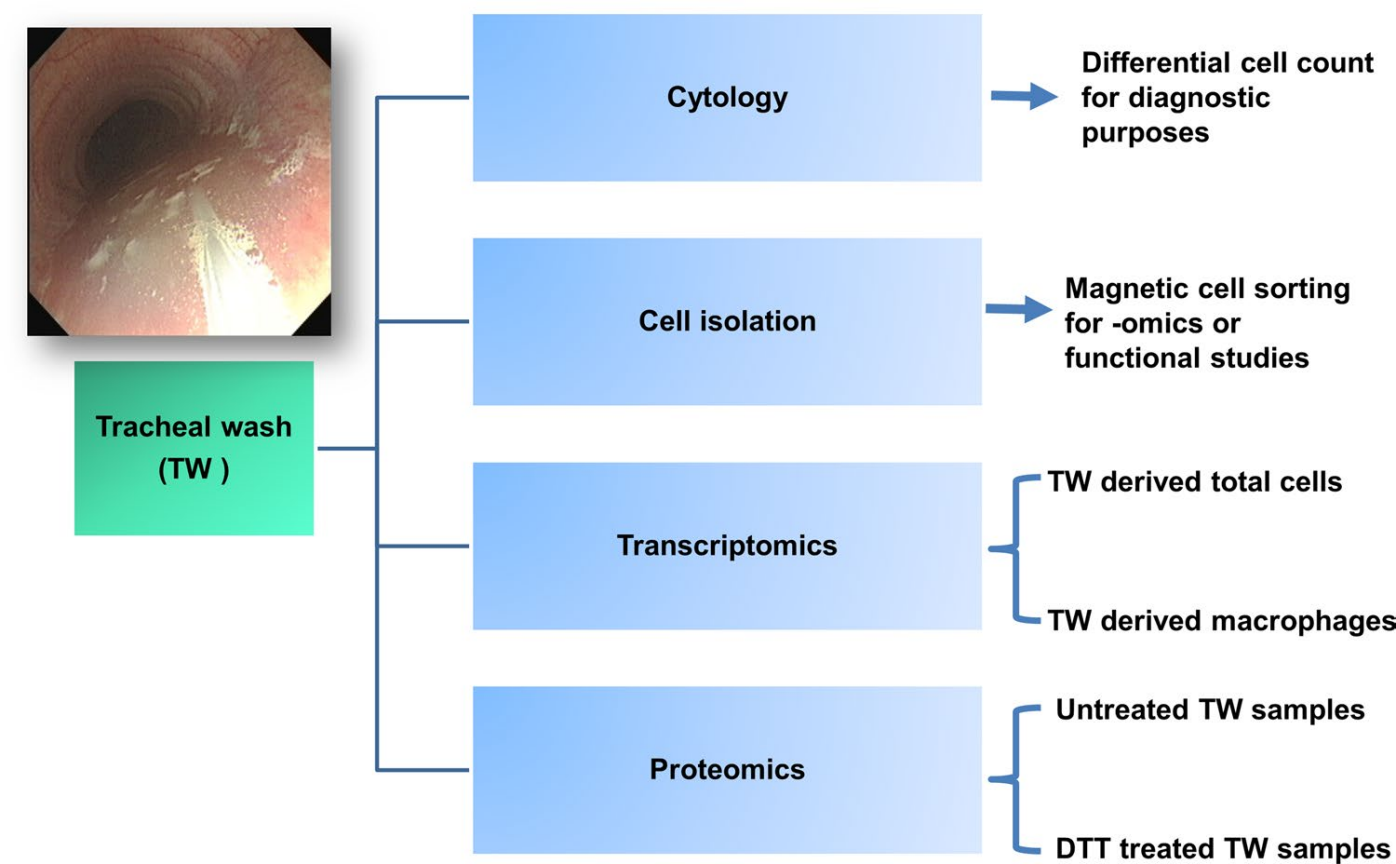
Multiple clinical and research applications can be investigated with a single tracheal wash sample. Endoscopic image is showing the sampling catheter (via the biopsy channel of the endoscope) within a “pool” of tracheal wash fluid at the level of the thoracic inlet. *TW* Tracheal wash, *DTT* dithiothreitol.

The aim of this project was to highlight the usefulness of TW samples for several biological applications (Fig. 1) and to establish the conditions for optimal RNA and protein isolation, in order to use for transcriptomic and proteomic analysis. Thus, TW samples from 39 Thoroughbred racehorses were collected. Furthermore, the proteomic analysis protocol was successfully applied to equine, murine and human BALF samples.

### Establishing a protocol for tracheal wash cell isolation

**Figure 2:**
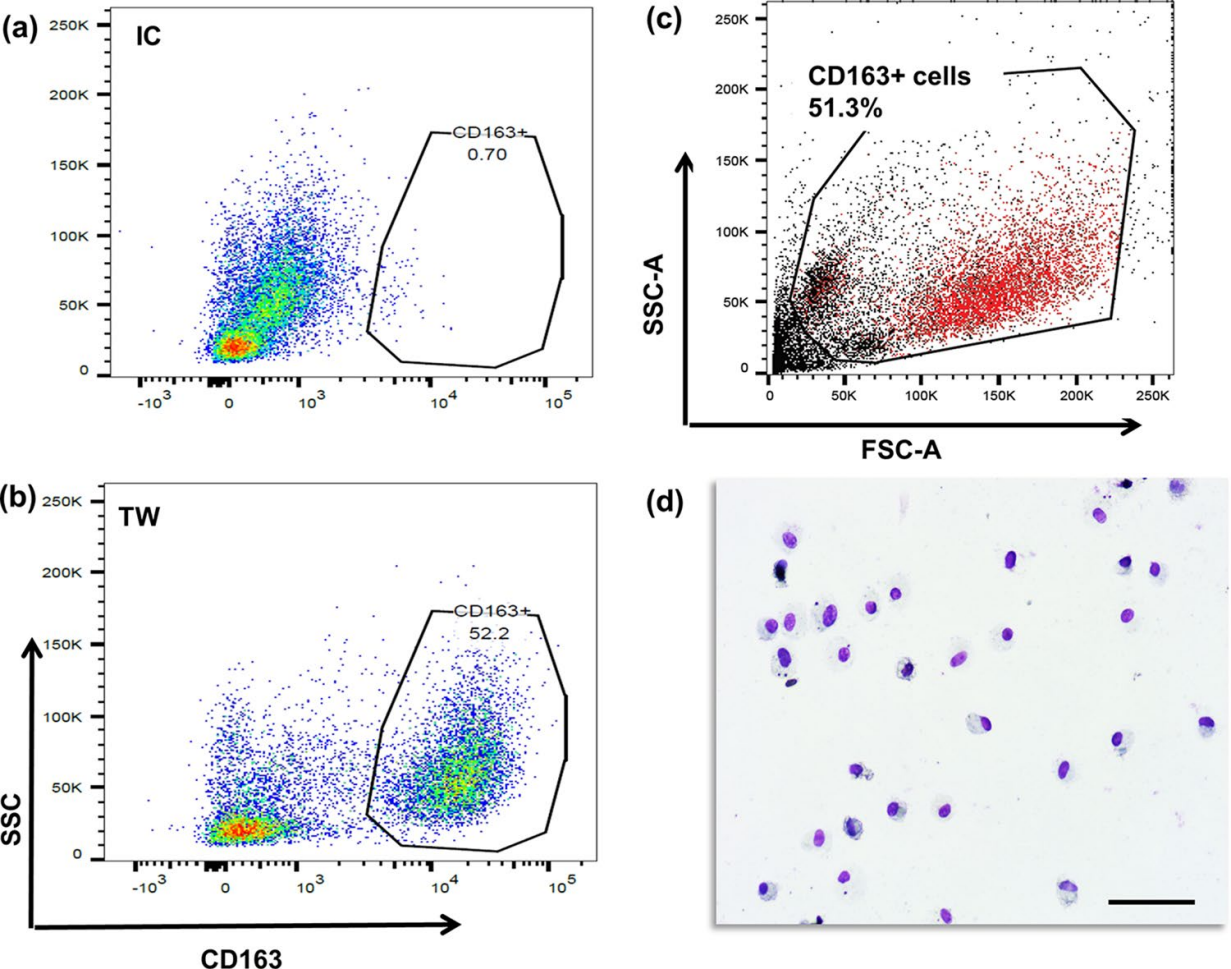
Analysis of tracheal wash derived macrophages. Flow cytometry results showing cross-reactivity of mouse anti-human CD163 antibody against equine tracheal macrophages. **(a)** Isotype control, **(b)** CD163 stained cells **(c)** overlay of CD163+ population on top of total cells. **(d)** Leishman stained cytospin preparations of CD163+ cells by light microscopy (× 20, scale bar = 50 μm). Data and image analysis was performed in FlowJo^®^ v10.5.3 https://www.flowjo.com/.

One aspect of the current project was the isolation of macrophages from TW samples. Analysis had confirmed cross-reactivity of an anti-human CD163 antibody, a classical marker for mature macrophages, and successful application of magnetic bead separation to dithiothreitol (DTT)-treated equine tracheal secretion samples. Flow cytometry analysis revealed that almost half of the cell population of TW samples were CD163 positive (Fig. 2a–c). Isolation of CD163 positive cells was also confirmed with light microscopy on cytospin slide preparations stained with Leishman stain (Fig. 2d).

The present study used a series of 39 horse-derived samples and an average of 5.9 ± 1.7 (± SEM) × 10^6^ cells were isolated from TW samples. RNA average yield extracted from the total cell population of TW samples was 244 ± 43 (± SEM)ng/µl. RNA yield concentration of CD163 positive cells was 43 ± 19 (± SEM) ng/µl. RNA integrity number (RIN) greater than 7 is recommended for RNAseq and qPCR analysis. RNA samples derived from the total population of TW-derived cells had an average RIN number of 7.92 ± 0.14 (± SEM), thus rendering samples suitable for sequencing or RNA analysis. Differential cell counts (DCC) of TW samples were as follows (mean ± SEM): 38.5 ± 3% macrophages (with no evidence of phagocytosed haemosiderin), 17.8 ± 2.8 macrophages with phagocytosed haemosiderin (haemosiderophages), 30.4 ± 1.7% lymphocytes, 13.3 ± 2.4% neutrophils, and 0.01 ± 0.02 eosinophils. The exclusion of epithelial cells is common practice in both equine and human pulmonology, unless they are being selectively harvested mainly for research purposes^26,27^. This is mainly due to the large variability in their relative proportion to other cell types, depending on factors such as coughing and collection technique and the potential to significantly impact the interpretation of inflammatory cell differential counts and skew data derived from down-stream analyses^27^.

**Figure 3:**
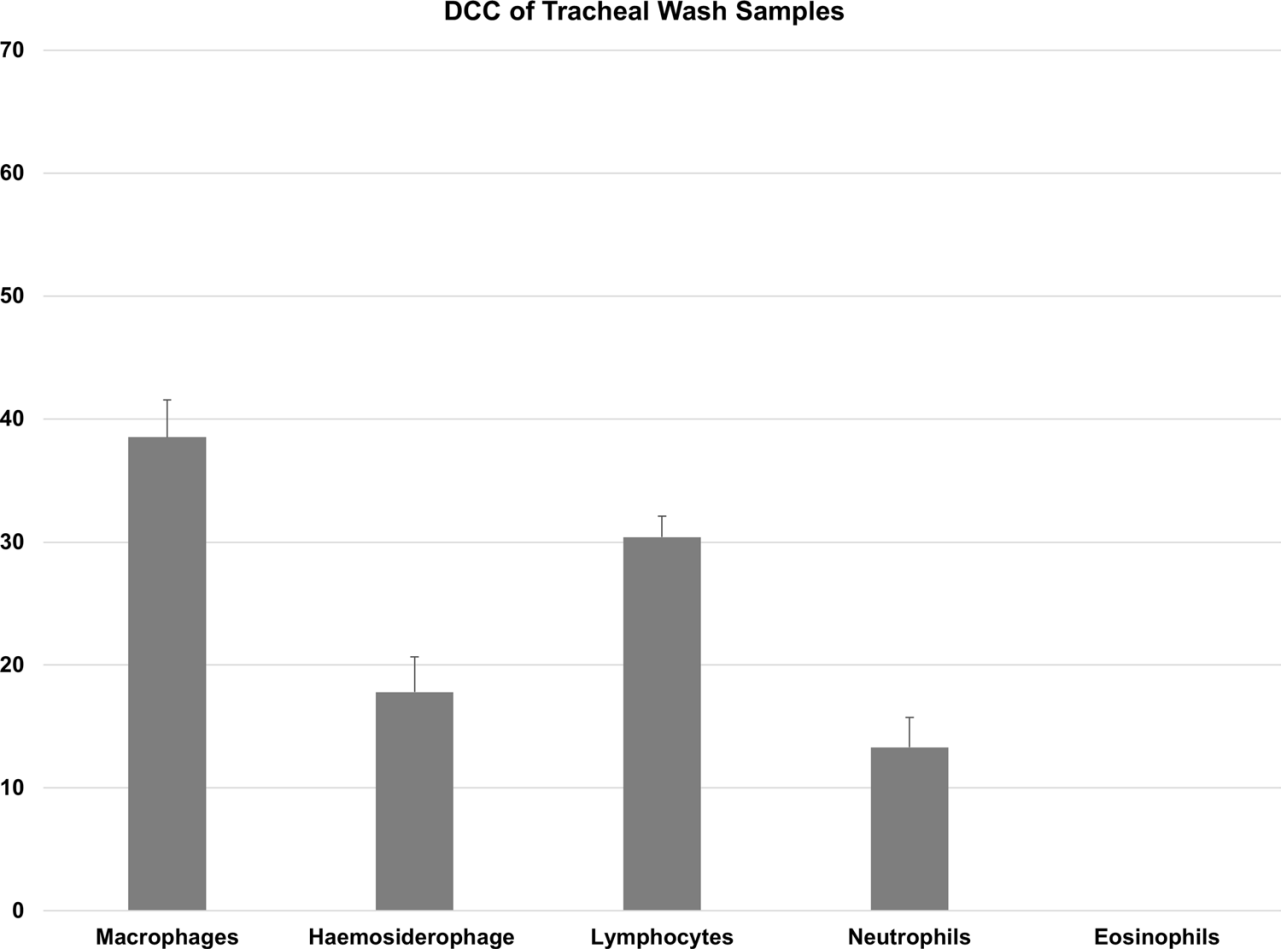
Differential cell count (mean + SEM %) of equine tracheal wash samples (n = 39). Differential leucocyte count (minimum of 200 cells) was performed and expressed as a percentage of total non-squamous and non-epithelial nucleated cells.

Results of the DCC are shown in Fig. 3. In agreement with the data derived from flow cytometric analysis (Fig. 2b) almost half the number of TW cells were macrophages (including haemosiderophages). Since all samples were collected from racehorses, the presence of haemosiderophages in these samples was unavoidable as almost all racing Thoroughbred horses will bleed into the airways; this condition is termed Exercise-Induced Pulmonary Haemorrhage^6^.

### Protein extraction of tracheal wash samples, has been successfully performed

In order to expand on the RNA studies, we planned to define the airway protein profiles (total proteome), thus revealing the mechanisms which may underpin any alterations in immune function. Protein extraction was successfully performed on TW samples from 39 horses. An average of 1.3 ± 0.2 (± SEM) mg protein has been isolated from 500 µl of TW per animal. Protein extraction of equine BALF samples resulted in 0.8 ± 0.07 (± SEM) mg protein isolated from 500 µl. The same protocol was successfully applied on the same volume of human (n = 3) and murine (n = 4) BALF samples, from which a protein yield of 0.6 ± 0.1 (± SEM) and 0.4 ± 0.1 (± SEM) mg total protein were isolated, respectively.

**Figure 4:**
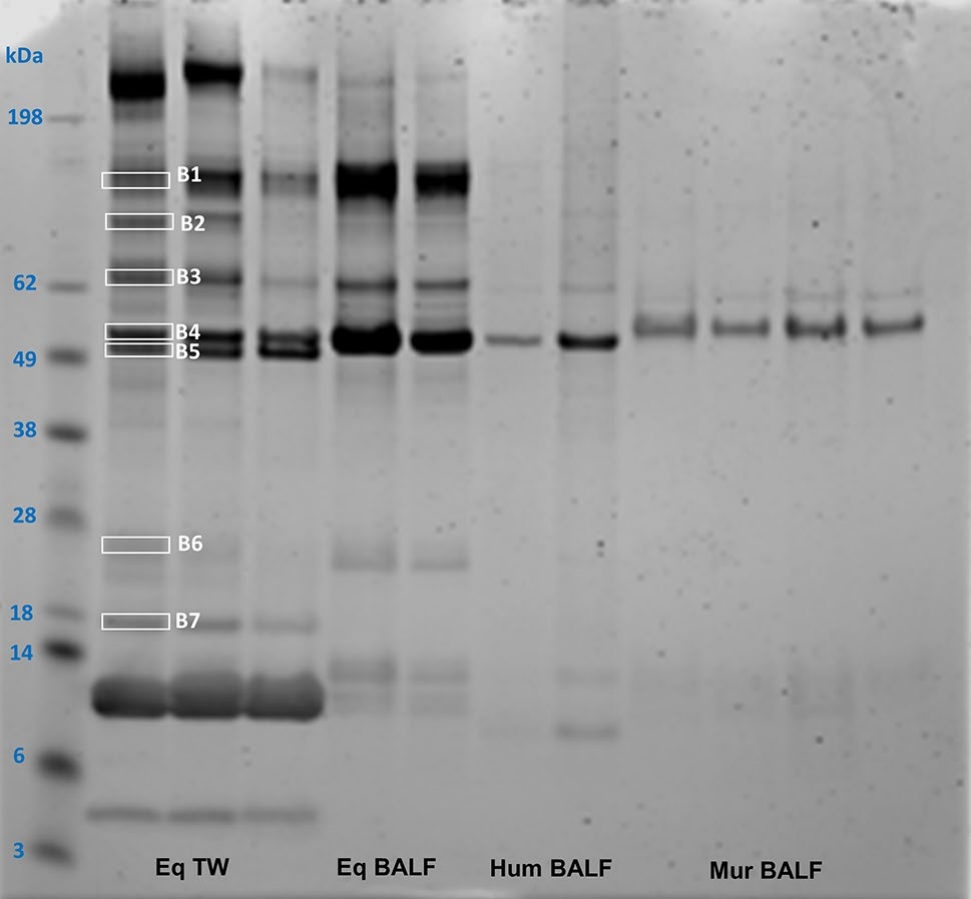
Comparison of detectable proteins isolated from equine tracheal wash and bronchoalveolar lavage samples of human, mice and horse. Lane 1 shows the protein ladder. Total protein stain of equine (Eq) TW samples (Lanes 2–4, 10 ug) and BALF samples (Lanes 5–6, 10 ug). Lanes 7–8 (5 ug) show total protein stain from human (Hum) BALF samples and lanes 9–12 (5 ug) contain total protein stain of four murine (Mur) BALF samples. Selected prominent bands isolated from the gel and analysed in the Mass Spectometry Facility are shown in white boxes (Lane 2, B1–B7). Note the increased diversity of proteins derived from equine TW samples compared to BALF. The stained gel was imaged using the LICOR Odyssey imager and the associated Image Studio Software (https://www.licor.com/bio/odyssey-fc/, https://www.licor.com/bio/image-studio-lite/, Version 5.2).

In order to visualise the total protein load, all samples were run on gradient gels and stained with instant blue protein stain. Figure 4 is representative of a gel stain of three equine TW, two BALF samples, as well as two human and four murine samples; a number of bands (e.g., B3, B5) showed a similar pattern in each species. However, it is important to consider the different protocols applied for sample collection between species, in particular if quantitative comparative analysis between species is to be performed. Protein samples were also submitted for proteomic analysis.

In order to investigate the variety of proteins present in the samples, selected prominent bands were isolated from the gel and analysed in the Mass Spectometry Facility at the Roslin Institute. Uniprot, a comprehensive resource for protein sequence and annotation data was used (https://www.uniprot.org/proteomes/UP000002281) for horse protein annotation.

Initial quality control analysis based on the staining patterns on gel clearly shows protein samples were intact and showed no signs of degradation, in spite of the sample preparation and addition of extraction buffer components directly into liquid biological samples. Also, a range of protein identities were revealed from a few bands analysed by LC–MS (Supplementary Data 1).

Furthermore, in an attempt to investigate whether DTT treatment has an effect on TW derived protein; a comparison was also made between DTT treated and untreated TW samples. The addition of DTT during sputum processing is widely used to release cells from mucus. However, it is reported that DTT treatment may result in decreased detection of inflammatory cytokines in human sputum compared to untreated samples^28^. In line with this, our results suggested that the yield of unique protein identifications from the untreated samples was double that derived from DDT treated samples (Supplementary Data 1). Therefore, we decided to proceed with proteomic analysis of TW samples that had not been treated with DTT. In total, 436 unique proteins were detected from all samples. Some example proteins detected in the TW samples are shown in Table 1. These are related to immune response and lung biology, including CD14, complement factors, immunoglobulins, surfactant proteins and mucins, suggesting that samples isolated and processed in this way are likely to be representative of the in vivo composition. The complete lists of mapped proteins are presented in the Supplementary Data 1.

**Table 1:** List of proteins related to immune response and lung biology derived from tracheal wash samples.

Protein ID	Protein annotation
F7AUX9	*Pulmonary surfactant-associated protein A* OS = Equus caballus OX = 9796 GN = SFTPA1 PE = 4 SV = 1
F7DJE3	*Surfactant protein D* OS = Equus caballus OX = 9796 GN = SFTPD PE = 4 SV = 1
P01958	*Haemoglobin subunit alpha* OS = Equus caballus OX = 9796 GN = HBA PE = 1 SV = 2
F6QDH8	*Alpha-amylase* OS = Equus caballus OX = 9796 GN = LOC100051073 PE = 3 SV = 1
F7BAY6	*Serum albumin* OS = Equus caballus OX = 9796 GN = ALB PE = 4 SV = 1
F6V5H1	*Immunoglobulin kappa constant* OS = Equus caballus OX = 9796 GN = IGKC PE = 4 SV = 1
H9GZN9	*Immunoglobulin heavy constant* mu OS = Equus caballus OX = 9796 GN = IGHM PE = 4 SV = 1
F6SP74	*Complement factor properdin* OS = Equus caballus OX = 9796 GN = CFP PE = 4 SV = 1
Q9GKX7	*Heat shock protein HSP 90-alpha* OS = Equus caballus OX = 9796 GN = HSP90AA1 PE = 2 SV = 2
F6R6Z6	*Epithelial cell adhesion molecule* OS = Equus caballus OX = 9796 GN = EPCAM PE = 4 SV = 1
F6WA57	*Lymphocyte cytosolic protein 1* OS = Equus caballus OX = 9796 GN = LCP1 PE = 4 SV = 1
F7CU94	*Lysozyme* OS = Equus caballus OX = 9796 GN = LYZ PE = 2 SV = 1
F7DBT2	*Complement C1q C chain* OS = Equus caballus OX = 9796 GN = C1QC PE = 4 SV = 1
F6VK89	*CD14 molecule* OS = Equus caballus OX = 9796 GN = CD14 PE = 4 SV = 1
F7CJ82	*Arginase* OS = Equus caballus OX = 9796 GN = ARG1 PE = 3 SV = 1
F7B5C4	*Vimentin* OS = Equus caballus OX = 9796 GN = VIM PE = 3 SV = 1
F6S981	*MHC class II antigen* OS = Equus caballus OX = 9796 GN = HLA-DRA PE = 2 SV = 1
F6QC83	*Mucin 5AC*, oligomeric mucus/gel-forming OS = Equus caballus OX = 9796 GN = MUC5AC PE = 4 SV = 1

**Figure 5:**
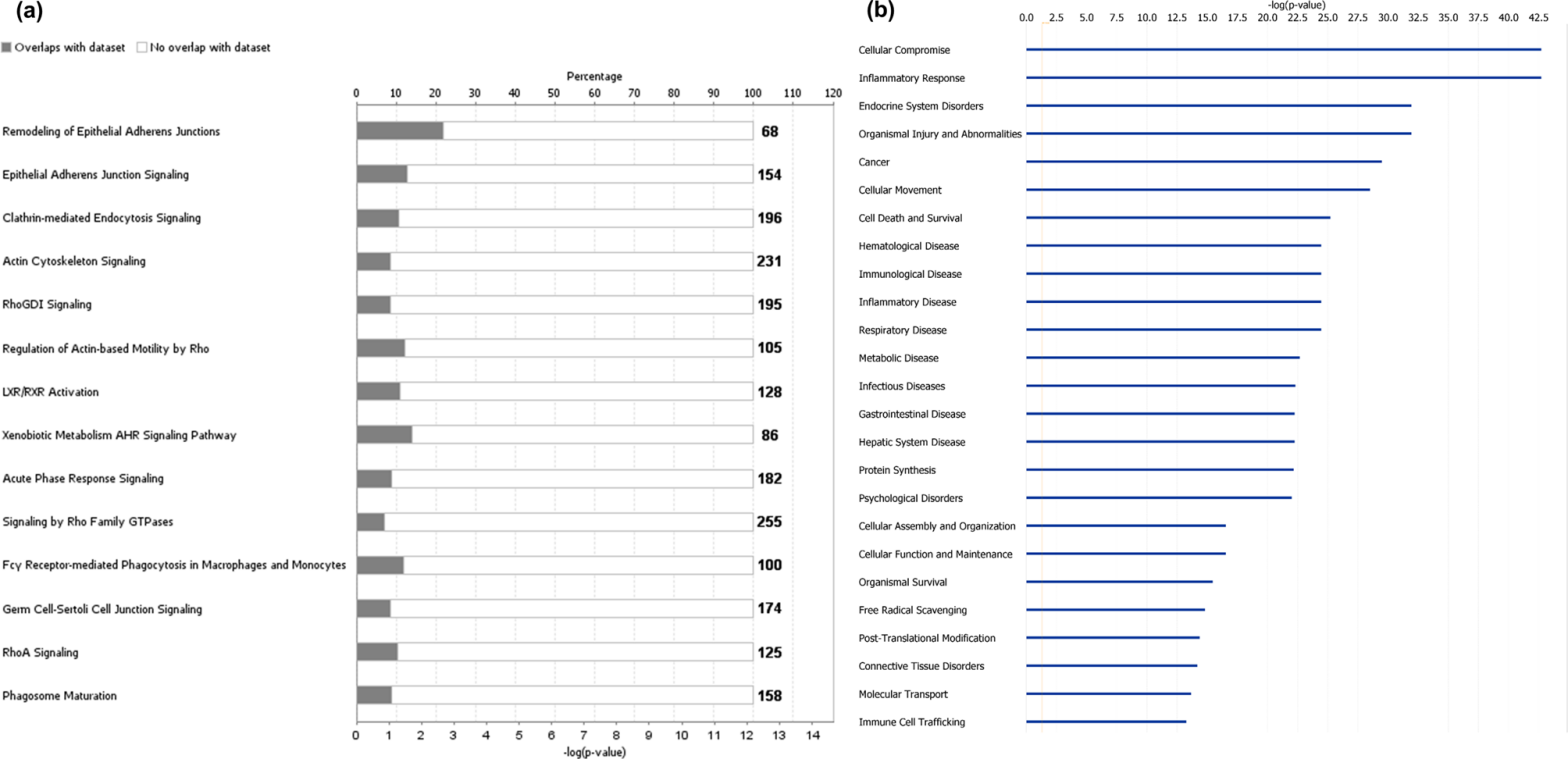
Functional analysis of the equine tracheal wash proteome. Ingenuity Pathway Analysis software was used to determine the **(a)** most enriched canonical pathways and **(b)** diseases and biofunctions associated with the protein list (IPA Spring 2021 release, https://www.qiagenbioinformatics.com/products/ingenuity-pathway-analysis)^30^.

**Figure 6:**
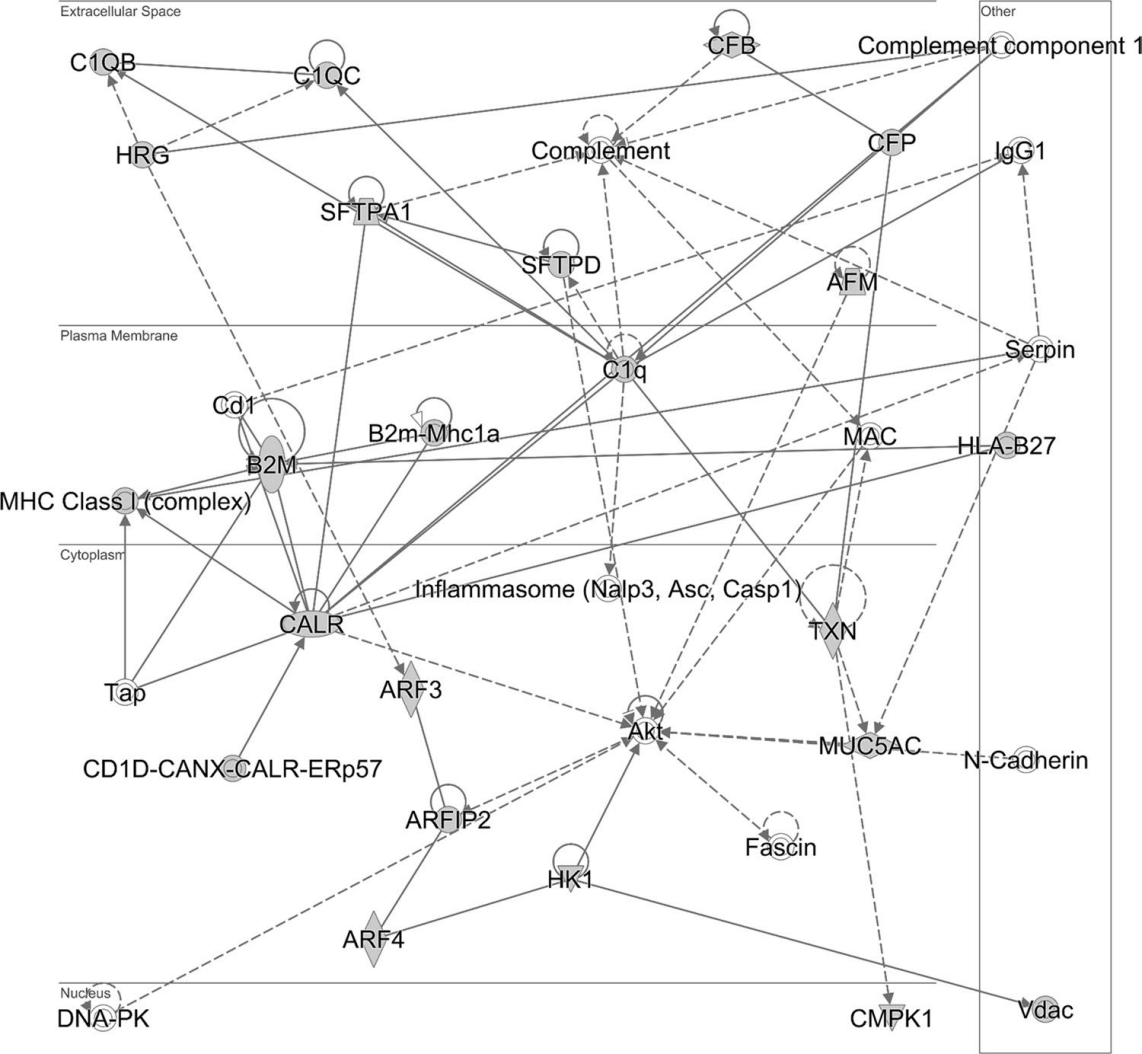
Pathway analysis identifies networks comprising immune-related proteins. Detected proteins are involved in “Cellular Function” and “Maintenance”, “Humoral Immune response” and “Inflammatory Response”. The network is displayed graphically as nodes (proteins) and edges (the biological relationships between nodes). Analysis was performed by the Ingenuity Pathway Analysis software (IPA Spring 2021 release, https://www.qiagenbioinformatics.com/products/ingenuity-pathway-analysis)^30^.

KEGG pathway enrichment analysis of the detected proteins was performed using the Database for Annotation, Visualization, and Integrated Discovery (DAVID) annotation software (https://david.ncifcrf.gov/)^29^. The detected protein list included proteins involved in metabolic pathways and immune response to infection (Supplementary Data 2). Ingenuity Pathway Analysis (IPA) was also used to functionally analyse the list of the detected proteins^30^. The main canonical pathways and diseases/biofunctions related to the protein list are shown in Fig. 5. The IPA-generated network (Fig. 6) contains most of the molecules related to “Cellular Function” and “Maintenance”, “Humoral Immune response” and “Inflammatory Response”.

## Discussion

To the best of our knowledge, this study has shown for the first time that it is feasible to carry out both transcriptomic and proteomic analysis on a single TW collected from equines, in this case UK national hunt Thoroughbreds. Such samples are routinely collected in veterinary practice, in contrast to the less readily available and more technically invasive BAL-derived samples, which have previously been favoured for transcriptomic and proteomic analyses^14,31,32^. Moreover, macrophages were successfully isolated from TW samples based on magnetic bead separation and isolated cells were deemed suitable for downstream transcriptomic analysis. Similar experiments have been performed on human isolated macrophages from induced sputum^33^ but this is the first time it has been carried out in the equine species.

Proteomic analysis revealed a wide range of proteins in the equine TW samples, many of which have already been detected and reported in previously published studies on human and equine BALF and human sputum^32,34–36^. Some of the detected proteins have previously been proposed as potential biomarkers for human lung cancer; these include carbonic anhydrase 1 (CA1), apolipoprotein A2 (APOA2), transthyretin (TTR), clusterin CLU, ceruloplasmin (CP) and gelsolin (GSN)^35^. In a similar context, TW proteomics can be envisioned in terms of biomarker screening for the development of ‘real’ biomarkers with potential future clinical utility in relation to two common respiratory diseases in horses; namely, equine asthma and exercise induced pulmonary haemorrhage^6,37^. Moreover, results may have translational application to human respiratory disorders, such as equine asthma or respiratory infections.

Several proteins were related to important biological processes of the respiratory system, such as cellular function/differentiation [annexin (ANXA4, ANXA7), cathepsin B (CTSB), transgelin 2 (TAGLN2)], immune defense/response [CD14 molecule (CD14), lysozyme (LYZ), complement components (C1QB, C1QC, C3, C4A, CFB)] and antigen processing and presentation [MHC class II DR alpha chain (DRA), beta-2-microglobulin (B2M), heat shock proteins (HSPA1A, HSP90AB1)]. Interestingly, MUC5AC, also detected in our analyses, has previously been shown to be one of the predominant mucins in airway secretions derived from healthy horses and humans. Both MUC5AC and MUC5B are products of epithelial goblet cells and submucosal glands^38–40^. Finally, consistent with the cytological profile of the TW samples derived from a Thoroughbred racehorse population and the high presence of haemosiderophages, several proteins related to haematopoietic homeostasis were also present in the equine TW samples; examples include haemoglobin, zeta (HBZ1) and glucose-6-phosphate dehydrogenase (G6PD). Even though there was no evidence of remarkable blood contamination within the samples, it is possible that some of these proteins may have originated from blood serum. This is a common problem, when performing proteomics from body fluids^41,42^, and should therefore be taken into consideration, especially when analysis is applied on animals with airway inflammation.

The non-bronchoscopic method of collecting human BALF is well recognised in paediatrics, where a formal bronchoscopy is both technically challenging and difficult to justify without clear clinical need. As it is relatively less invasive, this alternative technique has several advantages over formal bronchoscopy in the paediatric setting. Furthermore, it requires relatively minimal training, avoids the requirement for expensive equipment and is quick and well tolerated by both patients with and without chest infection. Its adoption is therefore suited to medical research in resource limited settings. Although previously used to successfully obtain dendritic cells from healthy children and those with RSV bronchiolitis^43^, this is the first report of its use to obtain samples for proteomic analysis.

In conclusion, we have developed a robust protocol for obtaining a high quantity and quality of RNA and protein for transcriptomic and proteomic analysis from a single equine TW sample. Our “proof of principle” results should facilitate future gene-expression and extensive proteomic studies offering deep proteome coverage utilizing TW samples. These technological advances have the real capacity to improve our understanding of the function of the pulmonary immune system of the horse. They may also permit a more definitive and comprehensive characterization of common diseases such as equine asthma, potentially revealing not only disease susceptibility-associated biomarkers but also novel therapeutic targets. Finally, comparative global analysis of gene/protein expression across airway derived samples between horse and human would allow further investigation of the horse as an appropriate candidate model for human disease, under the umbrella of the “One Health” concept.

## Methods

### Samples used in the study

#### Equine samples

Tracheal secretions were collected from 39 (38 male and 1 female) Thoroughbred horses [7 ± 0.2 years (mean ± SEM); range 4–12 years]. All horses were housed at the same racing yard and samples were obtained as part of a routine assessment of respiratory health (differential cytology). Residual sample was retained for an ongoing proteomic and transcriptomic study. Sample collection procedures were approved by the Veterinary Ethical Review Committee of the School of Veterinary Medicine, University of Edinburgh, following the relevant guidelines and regulations relating to the provisions of the Animals (Scientific Procedures) Act 1986. All methods reported are in accordance with ARRIVE guidelines (https://arriveguidelines.org)^44^. Informed consent was obtained from the trainer for the use of residual sample material. Four BALF samples were collected at the Royal (Dick) School of Veterinary Studies, University of Edinburgh. All horses were clinically healthy and treated according to standard welfare procedures.

#### Murine samples

Bronchoalveolar lavage fluids were generously provided by Prof Jurgen Schwarze (Medical Research Council Centre for Inflammation Research, University of Edinburgh, UK). Samples were collected from four wild type BALB/c female mice, aged 8–12 weeks, at four days following intranasal administration of UV treated Respiratory syncytial Virus (RSV) as described by Kast et al*.*^45^. These mice represented controls to compare against models of RSV infection for investigating the influence of RSV infection on tight junction integrity. All experimental procedures were carried out in accordance with ARRIVE guidelines (https://arriveguidelines.org)^44,45^. All experimental protocols were approved by the Ethical Review Committee of the Medical Research Council Centre for Inflammation Research, University of Edinburgh, UK, as previously described^45^. All methods were carried out in accordance with relevant guidelines and regulations of the Home Office regulations^45^.

#### Paediatric samples

Bronchoalveolar lavage fluid was collected from three children, with no evidence of lung disease, who were undergoing planned procedures under general anaesthetic. Children were attending for planned surgical procedures (inguinal hernia and hypospadias repair and developmental dysplasia of the hip repair) and were aged 6 months, 2 and 5 years old (2 female, 1 male). Children were sampled at the Royal Hospital for Sick Children Edinburgh as part of the Lower respiratory tract infection Kids Study approved by the South East Scotland Research Ethics Committee 02. Informed consent was obtained from appropriate adult with parental responsibility. The clinical study and all associated procedures were conducted within Good Clinical Practice guidelines and the regulations stipulated by the MHRA (Medicines Healthcare Regulation Agency).

### Sample collection

#### Equine samples

All TW samples were collected one hour following morning exercise (interval training) using a trans-endoscopic technique, as previously described^5^. Horses were restrained and a nose twitch applied when necessary. A 1500 mm working length, 9.2 mm outer diameter video endoscope (2.8 mm biopsy channel; Aohua, China), was passed via the ventral nasal meatus to the pharynx and then advanced into the trachea via the *rima glottidis*. Following assessment of the amount and nature of mucus deposits within the tracheal lumen, approximately 20 ml of sterile 0.9% saline at room temperature was instilled via a catheter passed via the biopsy channel of the endoscope at the proximal aspect of the trachea. The endoscope was then further advanced to the level of the thoracic inlet where the pool of instilled fluid had gravitated. As much fluid as possible was subsequently aspirated via the transendoscopic catheter. Samples were stored on ice and submitted for laboratory analysis at the Roslin Institute and the Royal (Dick) School of Veterinary Studies and processed within 4–5 h of collection. Three BALF samples were collected at the Royal (Dick) School of Veterinary studies from horses admitted to the hospital, as previously described^46^; these procedures were performed for diagnostic purposes, with residual BALF made available for this study with owner consent.

#### Murine samples

Bronchoalveolar lavage fluid was collected by injecting 1 ml of PBS containing protease inhibitor via tracheal cannula—650–900 µl of fluid being retrieved, as previously described^45^. BALF supernatant was then collected and stored at − 80 °C till further use.

#### Paediatric samples

The non bronchoscopic bronchoalveolar lavage protocol was adapted from the European Respiratory Society BAL guidelines for children^47^, by the specialist paediatric physiotherapist Andrea Wood and Dr Jonathan Twynam-Perkins to allow less invasive and technically simpler sampling of the bronchoalveolar space in children. While anaesthetised a suction catheter was inserted blindly down the endotracheal tube and 2 ml/kg of 0.9% sodium chloride instilled and around 1 ml/kg of fluid being retrieved. Following a short period of time, and without removing the suction catheter, sample was collected by applying 10-15kpa of suction to said catheter. Resultant BALF was spun at 400×*g* at 4 °C for 10 min, the supernatant collected and then frozen at − 80 °C for storage and further analysis.

### Total and differential cell count and total cell and macrophage isolation for equine tracheal wash samples

#### Differential cell count

An aliquot of 0.5 ml was submitted to the pathology lab at the Royal (Dick) School of Veterinary Studies for DCC analysis. Differential leucocyte count (minimum of 200 cells) was performed and expressed as a percentage of total non-squamous and non-epithelial nucleated cells. An aliquot was retained for cytological analysis as described previously^23,48^. Briefly, cell numbers were adjusted to 5 × 10^5^ cells/ml by the addition of a calculated volume of PBS. From this aliquot, 2 cytospin slide preparations were made per lavage [cytospined at 300 rpm (10 g) for 3 min] and stained (Leishman stain; L/1815L/PB05, Fisher Scientific, Leicestershire, UK) and a differential cell count calculated under light microscopy by counting 200 cells per animal.

Horses were considered free of inflammatory airway disorders based on the following DCC cut off values: neutrophils < 20%^4,5^.

#### Total cell isolation

First, a 1 ml aliquot of sample was immediately stored at − 80 °C for future proteomic analyses and the remaining sample incubated for 15 min at room temperature in 0.1% dithiothreitol (DTT) to depolymerize secreted mucin. Dithiothreitol has been demonstrated to cause no deleterious effects on human airway derived cells or interfere with surface marker measurements using flow cytometry^49^. Following gravity filtration through a 70 µm pore mesh filter, the sample was centrifuged at 400×*g* for 10 min at 4 °C. Supernatant was carefully removed, and the cell pellet resuspended in Dubelcco’s PBS, from which a total cell count (excluding squamous epithelial cells) and cell viability (Trypan Blue exclusion staining) was performed using a haemocytometer. Afterwards, the cell pellet was resuspended in 1 ml of Trizol and stored at − 80 °C for future RNA analysis.

#### Macrophage isolation

Following total cell isolation, immunomagnetic separation was used to isolate macrophages from DTT treated TW samples from 16 of the 39 animals, using magnetic beads coated with mouse anti-human CD163 antibody (GHI/61, BioLegend, cat no 333605), according to the manufacturer’s instructions. We have previously demonstrated cross-reactivity of CD163 antibody with equine macrophages/monocytes^23^. Briefly, the cells were first stained with a R-Phycoerythrin (PE)-conjugated CD163 primary antibody; subsequently, the cells were magnetically labelled with anti-PE MicroBeads (Miltenyi Biotec, cat no 130-105-639) before the cell suspension was loaded on a MACS^®^ LS Column (Miltenyi Biotec Ltd., cat n0 130-042-401), placed in the magnetic field of a MACS Separator. The magnetically labeled cells were retained within the column, while the un-labeled cells passed through. Following removal of the column from the magnetic field, magnetically retained cells were eluted as the positively selected cell fraction.

### Flow cytometry analysis of tracheal wash derived cells

Total TW cells were stained with anti-CD163 antibody against macrophages (BioLegend, Catalogue No. 333605). Cells were also stained with Zombie aqua dye (Biolegend, Catalogue No. 423101) to test for viability. A negative/isotype control was used to control for non-specific binding (PE Mouse IgG1, κ Isotype Ctrl, Biolegend, Catalogue No. 400111). Data were acquired on 10,000 live cell events and cells gated according to size (FSC-A: forward scatter) and granularity (SSC-A: side scatter) after removing artifacts, debris, doublet discrimination and dead cells. Data analysis was performed in FlowJo^®^ v10.5.3 (Tree Star, https://www.flowjo.com/).

### RNA analysis of equine tracheal wash derived cells

#### Total RNA extraction

Total RNA was extracted using a combination of Trizol reagent (Thermo Scientific™, 15596026) and an RNAeasy plus micro kit (Qiagen, cat no 74034), according to manufacturer’s instructions. Briefly, following removal of the supernatant, the remaining cell pellet was lysed by the addition of 1 ml Trizol Reagent. Subsequently, 0.2 ml 1-Bromo-3-chloropropane (BCP) (Sigma Aldrich, cat no B9673-200ML) was added per 1 ml of Trizol. The sample was shaken vigorously for approximately 30 s and left at room temperature for 5 min to allow complete dissociation of nuclear-protein complexes. The homogenate was then centrifuged at 18,000×*g* for 15 min at 4 °C resulting in the formation of a lower red phenol–chloroform phase, an interphase, and an upper colourless aqueous phase. The aqueous phase contained the RNA and had almost 50% of the volume of the Trizol used, plus the volume of the sample. Following transfer to a clean tube for the precipitation step, 0.5 ml of 70% ethanol was added, the sample stored for 2 h at − 20 °C and then transferred to an RNeasy spin column and centrifuged at 18,000×*g* for 5 min at 4 °C. Following centrifugation, the flow through was removed, the RNA washed once with RW1 buffer and DNA treatment was performed using the RNase-Free DNase Set (Qiagen, cat no 79254) according to the manufacturer’s instruction. Due to potential DNA contamination, this step was performed twice and samples were run through gDNA Eliminator Spin Columns twice after the elution step. Afterwards, the RNA membrane was washed with RW1, RPE and 80% ethanol. Finally, RNA was eluted in 20 µl RNase-free water and RNA samples were stored at − 80 °C until further use.

#### RNA quality assessment

RNA concentration and purity were measured using ND-1000 Nanodrop spectrophotometer (Thermo Scientific, Wilmington, USA) by measuring absorbance at 260 and 280 nm (A260, and A280 respectively). Purity of RNA was determined using the A260/A280 ratio. A ratio close to two was considered to be indicative of pure RNA. RNA integrity was confirmed with the High Sensitivity RNA ScreenTape system (Agilent Technologies). A RIN number greater than seven was considered appropriate for RT-qPCR and RNAseq analysis.

### Protein analysis

Briefly, an aliquot of 500 µl of untreated TW or BALF samples was homogenized in protein extraction buffer (100 mM Tris, pH 7.6 and 4%w/v SDS) + 1% Halt Protease Inhibitor Cocktail, EDTA-Free (Thermo Scientific, cat no 87785). Following homogenization, samples were centrifuged at 20,000×*g* for 20 min at 10 °C. The supernatant containing the solubilized protein was removed and stored at − 80 °C. Protein concentration of samples was determined using a Micro BCA Protein Assay Kit (Thermo Scientific™, cat no 23235) according to the manufacturer’s instructions. Finally, total protein analysis was carried out for quality control purposes and to determine equivocal protein load between samples. Samples were separated by electrophoresis on gradient gels (NuPAGE 4–12% Bis–Tris Protein Gels, 1.0 mm, 12-well, Fisher Scientific, cat no NP0322BOX) and stained with InstantBlue™ Protein Stain (Expedeon Ltd, cat no ISB1L) as previously described^50^. The stained gel was then imaged using the LICOR Odyssey imager to visualise and quantify the total protein load within each lane of the gel using the associated Image Studio Software (https://www.licor.com/bio/odyssey-fc/, https://www.licor.com/bio/image-studio-lite/, Version 5.2).

#### Trachea wash sample preparation for LC–MS

Sample preparation for LC–MS was carried out using S-Trap micro spin column digestion protocol (Protifi, Huntington, NY). Samples containing 5–10 μg of protein, were diluted to 50 μL with lysis buffer (5% SDS in 50-mM TEAB) and reduced with 20 mM Dithiothreitol at 95 °C for 10 min. After cooling to room temperature, samples were alkylated in the dark for 30 min with 40 mM of iodoacetamide. This mix was then acidified to 1% phosphoric acid and proteins were precipitated with 350 μl of binding buffer [90% methanol, 100-mM Triethyl ammonium bicarbonate (TEAB)]. This protein suspension was loaded onto S-Trap column columns and centrifuged at 4000×*g* for 30 s. After four washes with 125 μl of 50-mM TEAB, trypsin (0.5 μg in 50 mM TEAB) was added to the trap and incubated overnight. Peptides were then eluted with 80 μl of each of the following: 50-mM TEAB, 0.2% formic acid (FA), and 50% acetonitrile in 0.2% FA with centrifugation at 1000 g after each elution step. All elution fractions were pooled and dried under vacuum and cleaned up by stage-tipping^51^.

#### LC–MS analysis on tracheal wash samples

Nanoflow LC–MS/MS was performed on a micrOTOF-II mass spectrometer (Bruker, Germany)^52^ coupled to a RSLCnano LC system (Thermo) following earlier method^53^ with minor modifications. Raw spectral data were processed with DataAnalysis (Bruker) software and the resulting peak lists were searched using Mascot 2.4 server (Matrix Science) against Uniprot horse sequence database (Uniprot ID UP000002281), containing 44,485 entries^52^. Precursor and fragment ion mass tolerance were set at 25 ppm and 0.06 Da respectively and false discovery rate was set at < 1% for peptide IDs after searching decoy databases.

### Protein function annotation and pathway analysis

As previously described^54^, identification of enriched KEGG pathways and biological processes in the protein list was performed with Database for Annotation, Visualization and Integrated Discovery (DAVID) software (v6.8)^29,55,56^. KEGG pathway database contains regulatory and metabolic pathways, representing global knowledge on molecular interactions and reaction networks. Pathway analysis was performed using Ingenuity Pathway Analysis (*IPA Spring 2021 release,* https://www.qiagenbioinformatics.com/products/ingenuity-pathway-analysis)^30^ to infer the functional roles and relationships of the detected proteins.

## Supplementary Information

Supplementary Information.

Supplementary Data 1.

Supplementary Data 2.

Supplementary Figure S1.
