# Supplementary material for: Application across species of a one health approach to liquid sample handling for respiratory based -omics analysis
Source: Sci Rep. 2021 Jul 12;11:14292. doi: 10.1038/s41598-021-93839-9 (PMC8275668; doi:10.1038/s41598-021-93839-9)
Supplement: Supplementary file 1 — Supplementary Information. [file 41598_2021_93839_MOESM1_ESM.docx]

**Additional information**

**Supplementary Information:**

**Supplementary Data 1 -** Complete lists of mapped proteins

**Supplementary Data 2 -** KEGG pathway and Gene Ontology enrichment analysis of the detected proteins
