# Supplementary figures and images for: Application across species of a one health approach to liquid sample handling for respiratory based -omics analysis
Source: Sci Rep. 2021 Jul 12;11:14292. doi: 10.1038/s41598-021-93839-9 (PMC8275668; doi:10.1038/s41598-021-93839-9)

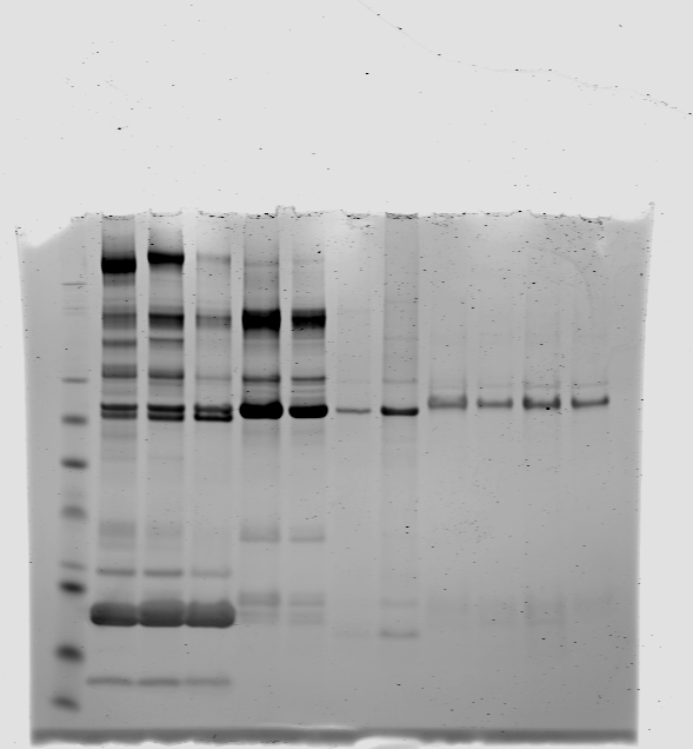

Supplement: Supplementary file 4 — Supplementary Figure S1. [file 41598_2021_93839_MOESM4_ESM.tif]
